# Persisting Social Participation Restrictions among Former Buruli Ulcer Patients in Ghana and Benin

**DOI:** 10.1371/journal.pntd.0003303

**Published:** 2014-11-13

**Authors:** Janine de Zeeuw, Till F. Omansen, Marlies Douwstra, Yves T. Barogui, Chantal Agossadou, Ghislain E. Sopoh, Richard O. Phillips, Christian Johnson, K. Mohammed Abass, Paul Saunderson, Pieter U. Dijkstra, Tjip S. van der Werf, Ymkje Stientstra

**Affiliations:** 1 University of Groningen, University Medical Center Groningen, Department of Internal Medicine/Infectious Diseases, Groningen, The Netherlands; 2 Programme National de Lutte contre la Lèpre et L'Ulcère de Buruli, Ministère de la Santé, Cotonou, République du Bénin; 3 Kwame Nkrumah University of Science and Technology, School of Medical Sciences, Department of Medicine, Kumasi, Ghana; 4 Fondation Raoul Follereau, Cotonou, République du Bénin; 5 Agogo Presbyterian Hospital, Agogo, Ghana; 6 American Leprosy Missions, Greenville, South Carolina, United States of America; 7 University of Groningen, University Medical Center Groningen, Department of Rehabilitation and Department of Oral and Maxillofacial Surgery, Groningen, The Netherlands; University of Tennessee, United States of America

## Abstract

**Background:**

Buruli ulcer may induce severe disabilities impacting on a person's well-being and quality of life. Information about long-term disabilities and participation restrictions is scanty. The objective of this study was to gain insight into participation restrictions among former Buruli ulcer patients in Ghana and Benin.

**Methods:**

In this cross-sectional study, former Buruli ulcer patients were interviewed using the Participation Scale, the Buruli Ulcer Functional Limitation Score to measure functional limitations, and the Explanatory Model Interview Catalogue to measure perceived stigma. Healthy community controls were also interviewed using the Participation Scale. Trained native interviewers conducted the interviews. Former Buruli ulcer patients were eligible for inclusion if they had been treated between 2005 and 2011, had ended treatment at least 3 months before the interview, and were at least 15 years of age.

**Results:**

In total, 143 former Buruli ulcer patients and 106 community controls from Ghana and Benin were included in the study. Participation restrictions were experienced by 67 former patients (median score, 30, IQR; 23;43) while 76 participated in social life without problems (median score 5, IQR; 2;9). Most restrictions encountered related to employment. Linear regression showed being female, perceived stigma, functional limitations, and larger lesions (category II) as predictors of more participation restrictions.

**Conclusion:**

Persisting participation restrictions were experienced by former BU patients in Ghana and Benin. Most important predictors of participation restrictions were being female, perceived stigma, functional limitations and larger lesions.

## Author Summary

Disabilities among Buruli ulcer patients remain a problem. Previous studies revealed contractures, deformities and functional limitations in daily life after treatment. According to the International Classification of Functioning, Disability and Health, disabilities occur not only at the physical and activity level but at the participation level (participation restrictions) as well. The latter are the social consequences of the disease such as problems in relationships, going to festivals and visiting public places. This study focused on participation restrictions by using the Participation Scale among former Buruli ulcer patients and healthy persons residing in two areas endemic for Buruli ulcer in Ghana and Benin. This study showed that almost half of the former Buruli ulcer patients encountered problems in social life, especially related to employment. In addition, the results suggest that being female, perceived stigma, functional limitations and a larger lesion (category II) predict participation restrictions. These findings indicate that rehabilitation programs should not only focus on physical disabilities but also on participation after completion of medical treatment.

## Introduction

Buruli ulcer (BU) is a skin condition caused by *Mycobacterium ulcerans*, which is the third most prevalent mycobacterial disease in immuno-competent humans, after the diseases caused by *Mycobacterium tuberculosis* and *Mycobacterium leprae* [1]. BU presents as a small nodule or a plaque sometimes accompanied by edema. At a later stage, the lesion breaks open with ulceration typically presenting with undermined edges [2]. The World Health Organization (WHO) has classified lesions as category I: lesions cross-sectional diameter of less than 5 cm; as category II: lesions of 5–15 cm and category III: lesions of >15 cm; category III also includes lesions on important sites (for example eyes) and multiple lesions. The exact mode of transmission remains unclear, though it is generally accepted that infection is associated with living close to stagnant water [3]. BU has been found in more than 30 countries predominantly with tropical or subtropical climates; the most burdened region is West Africa. In 2011, Côte d'Ivoire, Ghana and Benin reported the highest numbers of new cases [4]. In Benin, the prevalence varies from 5.4 cases/10,000 to 60.7/10,000 inhabitants depending on altitude of villages [5] while the national BU prevalence in Ghana is 20.7 cases/100,000 inhabitants [6]. Since 2005, standard medical treatment entails antimicrobial therapy sometimes complemented with surgery [7].

Prevention of Disability (POD) programs have been developed by the WHO, which are implemented in endemic countries to reduce disabilities. Essential components are wound management, and positioning and mobilization of the affected extremity. Nevertheless, studies have revealed that people still develop physical disabilities such as scarring, contractures, deformities, and sometimes require amputation [8], [9] or are otherwise left with functional limitations [10], [11]. Not only may BU lead to physical consequences, but also stigmatization is perceived by former BU patients, even years after healing [12]. Magico-religious ideas on the cause of BU, fear of contracting the disease and its visible signs are suggested to be the most important distinctive features of this stigma [13]. In other stigmatized health conditions such as leprosy and leishmaniasis, participation restrictions in social life after treatment are common [14]–[18]. Participation restrictions are defined as ‘any problem an individual may experience in involvement in life situations' [19]. For example, a person may encounter restrictions related to employment, meeting new people, visiting public places or attending social events in the community. Participants of a qualitative study have expressed that scarring and physical disabilities as a result of BU disease may result in problems with marriage and employment [20]. In addition, community members expressed persisting negative attitudes towards BU patients resulting in social exclusion as victims are believed to have no social responsibilities and should be restricted in attending social events [21]. Social problems are of particular importance because of their impact on a person's well-being and quality of life [22]. The aim of this study was to explore participation restrictions among former BU patients and to gain insight into the factors that predict participation restrictions.

## Methods

### Study population

From January to October 2012 data for this cross-sectional study were collected in Ghana and Benin. Eligible for inclusion were former BU patients aged at least 15 years, who were treated between 2005 and 2011, and whose treatment was completed at least 3 months before the study commenced. Medical records of the Centre de Dépistage et de traitement de l'Ulcère de Buruli de Lalo in Benin and Agogo Presbyterian Hospital in Ghana were screened for potential participants. In the absence of an address system and with no phone numbers recorded, potential participants had to be sought in the villages. In Benin a high number of potential participants were found, and therefore primary health care posts surrounding the hospital were chosen as study sites. Posts were selected if a high number of cases was found, from the medical records, in the catchment area of the post and if they were relatively easy to access (in terms of distance and road circumstances). In Ghana, former BU patients who participated in another follow-up study of the BURULICO trial in Ghana [23] were excluded. Healthy community controls without any history of BU or without a visible disability were recruited from villages located in the study area. In both countries, we aimed to include at least 50 healthy controls. Community controls representing the same age (+5/−5 years), female/male ratio and geographical location as the former BU patients were recruited.

### Questionnaires

#### Socio-demographic factors

To obtain information on socio-demographic variables a questionnaire was developed containing questions on current medical status, age, sex, location of residence, educational level, occupation and living situation. History of BU disease was traced from the BU01 forms and/or medical records. A visible deformity was established by the interviewers before the interview.

#### Participation restrictions

Participation restrictions were assessed with the Participation Scale (P-scale). The P-scale consists of 18 items covering eight major life domains distinguished by the International Classification of Functioning, Disability and Health (ICF) [24]. The instrument is generic and uses a peer-comparison to diminish cultural influences. The participants are asked to compare themselves to someone who is similar in all social-cultural, economic and demographic aspects, except for the disease or disability. Each item entails an objective question (responses; yes, no, sometimes or not specified, not answered or irrelevant) and a subjective question to grade participation restrictions (responses; ‘no problem’ (1)’, ‘small problem’ (2), ‘medium problem’ (3) and ‘a large problem’ (5)). Item scores are summed for the total score (range 0–90). Higher scores signify more participation restrictions. The cut-off value, based on local reference data, for participation restrictions was established to be 12 in the development study [24]. We decided to calculate a cut-off value, an approach which was recommended for this instrument, which was developed and validated in other cultural contexts [24].

#### Functional limitations

The Buruli Ulcer Functional Limitation Score (BUFLS) [25], [26] was employed to establish perceived functional limitations. Questions aim to gain insight into patients' perception of ability in 19 daily tasks divided into four groups: food preparation, personal care, daily work activities and mobility. The BUFLS uses an ordinal response scale; 0 points reflect an activity is performed without problems, 1 point indicates an activity is performed with difficulty and 2 points denote that an activity is impossible to perform. The total score, ranging from 0 to 100, is the sum score divided by the maximal score applicable for the patient and multiplied by 100. Higher scores denote a higher level of functional limitations.

Perceived stigma: A subset of questions (15 in total) of the Explanatory Model Interview Catalogue (EMIC) [27] which has been used earlier [13], [28], was used to explore perceived stigma [29]. Response options are ‘yes’ (3 points), ‘possibly’ (2 points), ‘uncertain’ (1 point) and ‘no’ (0 points). Each question contributes equally to the sum score. Sensitive questions concerning marriage, sexual functioning and fertility were not asked to participants below the age of 20 years. To compensate for items not asked, the total stigma score was calculated as the percentage of the maximum score a participant could receive on the questions applicable for that participant. Higher scores are indicative of a higher level of perceived stigma.

### Translation

Procedures regarding translation of the P-scale are extensively described elsewhere [30] briefly summarized the scale was translated and back translated into Twi (language in Ghana) and French (Benin).

### Procedures

Before data collection, in each country two native language speaking interviewers participated in a training to prevent bias during the interview. The training was provided using the available manuals; the Participation Scale Users Manual (version 6.0) and the BUFLS Manual (2012). During data collection regular discussions were held to reveal difficulties encountered during interviewing. During the interviews no specific problems were encountered with understanding the peer comparison. Former patients with BU were identified with either the assistance of a BU coordinator, a health care worker, or one of the local community volunteers. If eligible former BU patients could not be found or appeared not to be in the village, a second visit was planned to ask for study participation. To ensure privacy during the interviews, private quiet places were used to conduct the interview.

### Ethical consent

Ethical approval was granted by the Medical Ethical Review Committees of the Kwame Nkrumah University of Science and Technology, School of Medical Sciences, Komfo Anokye Teaching Hospital in Ghana (ref: CHRPE/RC/127/12) and the Ministry of Health in Benin (ref: N01961/MS/DC/SGM/DRF/SRAO/SA). Adult participants provided written informed consent. A parent or guardian of any child participant provided informed consent on their behalf.

### Statistical analysis

Data analyses were performed with Statistical Package for the Social Science (SPSS) version 20.0. The cut-off for the P-scale scores was determined calculating the 95th percentile of the P-scale sum scores of healthy community controls [24]. Two outliers in Ghana were removed for this analysis. The resulting cut-off was 16, indicating that participants with scores up to 16 were categorized as not having participation restrictions and participants with scores 17 or higher were categorized as having participation restrictions. Basic features of the data were analyzed using descriptive statistics. As appropriate, Pearson's chi-square test, Fisher's exact test, Mann-Whitney U test, Kruskal-Wallis test and Spearman Ranks correlation were performed to compare for differences in socio-demographic factors and clinical aspects across countries as well as for univariate associations with P-scale sum scores. Factors significantly related (*P*<0.1) to P-scale sum scores were entered as potential predictors of participation restrictions in a linear regression analysis. Residuals were checked for a normal distribution. The variable: ‘visible deformity‘ was removed for analysis because of missing values (*n* = 41). Predictors were removed from the model when removal criterion was met (*P*>0.1). Interaction terms (country x sex, sex x stigma scores, sex x age, and country x stigma score) were explored, also using differences found in the previous analysis [30] between Ghana and Benin. For interpretability, age was centered at 15 years as minimum age of the BU patients was 15 years of age.

## Results

### Population

In total 121 patients were treated for BU in Agogo Presbyterian Hospital in Ghana between 2005 and 2011 of which 46 could not be found. Reasons were unknown addresses (20), unclear information on name or location (16), had died (6) or were not traced (4) resulting in participation of 75 former patients with BU in Ghana. In Benin, a total of 4 village health centers were visited resulting in 255 patients treated for BU between 2006 and 2011. In total 68 former patients with BU could be traced. Reasons why patients could not be found were not recorded. Significant differences between Ghana and Benin were found in length of time since start of treatment, type of treatment, lesion size, type of lesion, visible deformity, profession, and living situation (Table 1).

**Table 1:** Characteristics of former BU patients.

Variables		Ghana (*n* = 75)	Benin (*n* = 68)	*P*-value
Age at time of inclusion, median (IQR)		27 (19;33)	25 (18;43)	.791*
Sex (male, %)		33 (44)	31 (46)	.859‡
Length of time since start treatment, median in months (IQR)		24 (15;37)	62 (33;71)	<.001*
Type of treatment	Antibiotic treatment, *n* (%)	55 (73)	29 (43)	<.001‡
	Antibiotics and surgery, *n* (%)	20 (27)	39 (57)	
Nr. of lesions, *n* (%)	1	68 (91)	61 (91)	.938‡
	>1	7 (9)	6 (9)	
Lesion size, *n* (%)	Category I	56 (82)	36 (53)	.001‡
	Category II	8 (12)	19 (28)	
	Category III	4 (6)	13 (19)	
Type of lesion	Nodule	31 (41)	2 (3)	<.001‡
	Plaque/edema	19 (26)	18 (26)	
	Ulcer	25 (33)	48 (71)	
Joint involved (yes, %)		24 (36)	27 (42)	.455‡
Visible deformity (yes, %)		1 (2)	17 (32)	<.001‡
Profession, *n* (%)	Employed/student	74 (99)	60 (88)	.010‡
	Unemployed	1 (1)	8 (12)	
Living situation, *n* (%)	Living with nuclear family	30 (40)	17 (25)	.011‡
	Living with extended family	41 (55)	51 (75)	
	With others	4 (5)	0	

*Mann Whitney U test, ‡Pearson chi-square or Fisher's exact test as appropriate.

### Profile of participation restrictions

**Figure 1:**
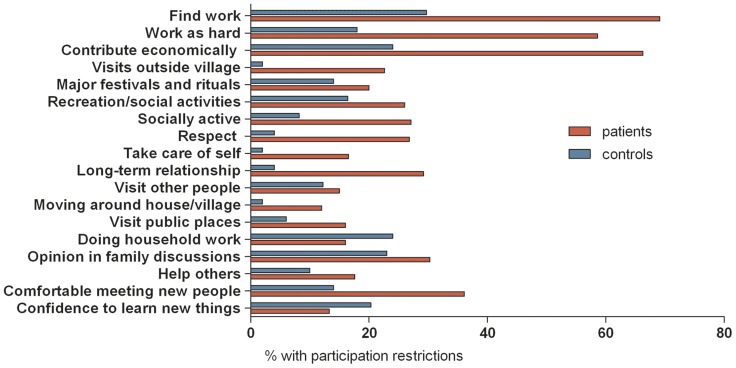
Profile of participation restrictions of patients and controls in Ghana. Red bar: patients. Blue bar: healthy community controls.

**Figure 2:**
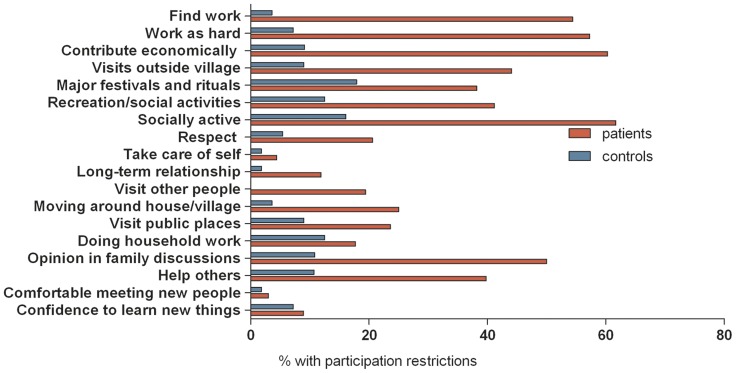
Profile of participation restrictions of patients and controls in Benin. Red bar: patients. Blue bar: healthy community controls.

Using a cut-off value of 16, in total, 67 (47%) former patients with BU experienced participation restrictions (median 30, IQR; 23; 43) while 76 indicated no participation problems (median 5, IQR; 2;9). Median P-scale sum scores of the former BU patients were similar in Ghana and Benin (Ghana: median 13, IQR; 5;29, Benin: median 13, IQR; 4;30). Across both countries, the most frequently reported problems among former BU patients were related to employment. In addition, in Ghana former BU patients experienced mainly problems with meeting new people, giving their opinion in family discussions, long-term relationships, being socially active, respect, and recreational and social activities. In Benin, former BU patients experienced mainly problems with being socially active, giving their opinion in family discussions, going for visits outside the village, recreational/social activities, helping others, and attending major festivals and rituals. Patients in Ghana had higher scores on each item compared to the healthy community controls, except for doing household work and confidence to learn new things. In addition healthy community controls in Ghana had higher levels of participation restrictions compared to healthy controls in Benin (Figure 1 and 2).

To illustrate, a former BU patient expressed her aspiration to be the teacher of a local woman's group, but because of BU she could not effectively organize the group. Another patient expressed his wish to be the leader of his political party in the future but he did not have enough money and because of the condition he could not succeed.

### Basic factors, functional limitations, perceived stigma and participation restrictions

In Benin, women, or patients with more than 1 lesion, a visible deformity or larger lesions scored significantly higher on the P-scale (Table 2). Furthermore, a median EMIC score of 19.5 (IQR; 10;36) was reported; women scored a median score of 19 (IQR; 9;36) and men 21.4 (IQR; 11;37). The median BUFLS was 7.9 (IQR; 0;20) and women (median 18, IQR; 3;24) scored significantly (*P* = .009) higher compared to men (median 0, IQR; 0;16). In Ghana, a median EMIC score of 20 (IQR; 13;53) was found; women scored a median score of 21.1 (IQR; 9;54) and men 20 (IQR; 13;54). The median BUFLS was 6.7 (IQR; 0;16) and women (median 11.8, IQR; 3;19) scored significantly (*P* = .043) higher compared to men (median 2.6, IQR; 0;13).

**Table 2:** Results of univariate analysis of variables among former BU patients associated with participation restrictions.

		Ghana			Benin			Total		
		Restricted, *n* (%)	Median P-scale score (IQR)	*P*-value	Restricted, *n* (%)	Median P-scale score (IQR)	*P*-value	Restricted, *n* (%)	Median P-scale score (IQR)	*P*-value
Sex	Women	22 (52)	7.5 (6;31)	.308*	22 (60)	23 (9;40)	.001*	44 (56)	19 (7;33)	.001*
	Men	14 (42)	12 (3;27)		9 (29)	7 (3;17)		23 (36)	9 (3;24)	
Nr. of lesions	1	33 (49)	2.5 (5;29)	.597*	26 (43)	13 (4;25)	.050*	59 (46)	13 (4;28)	.096*
	>1	3 (43)	14 (11;35)		5 (83)	33 (17;47)		8 (62)	22 (12;37)	
Type of treatment	Antibiotic treatment	24 (44)	12 (4;28)	.356*	10 (35)	9 (4;25)	.503*	34 (41)	10.8 (4;27)	.246*
	Antibiotic and surgery	12 (60)	20 (6;32)		21 (54)	17 (5;32)		33 (56)	19 (6;32)	
Lesion size	Category I	22 (39)	10 (4;24)	.159§	12 (33)	8 (3;25)	.024§	34 (37)	9.5 (3;25)	.005§
	Category II	5 (63)	8.5 (6;61)		10 (53)	17 (5;24)		15 (56)	17 (5;42)	
	Category III	3 (75)	6.5 (9;34)		9 (69)	31 (13;51)		12 (71)	29 (13–36)	
Type of lesion	Nodule	15 (48)	14 (8;30)	.439§	1 (50)	15.6 (3;-)	.428§	16 (49)	14 (7;29)	.807§
	Plaque/edema	11 (58)	21 (6;30)		6 (33)	5 (3;42)		17 (46)	11 (3;31)	
	Ulcer	10 (40)	10 (2;25)		24 (50)	16 (6;30)		34 (47)	13 (5;25)	
Joint involved	No	17 (40)	10 (5;27)	.171*	14 (38)	8 (3;27)	.108*	31 (39)	9.5 (3;27)	.056*
	Yes	16 (67)	20 (9;32)		13 (48)	15 (6;32)		29 (57)	18 (8;32)	
Visible deformity	No	20 (42)	10 (4;25)	.082*	13 (36)	8 (3;25)	.060*	33 (39)	8.5 (3;25)	.023*
	Yes	1 (100)	-		9 (53)	17 (11;39)		10 (56)	19 (11;45)	
Profession	Employed/student	36 (49)	13.5 (6;29)	.213*	28 (47)	14 (4;30)	.841*	64 (48)	13.5 (5;29)	.790*
	Unemployed	0	-		3 (38)	13.3(5;34)		3 (33)	13 (3;30)	
			Correlation			Correlation			Correlation	
Age at time of inclusion			0.21	.073‡		0.31	.012‡		0.25	.003‡
Length of time in months since start treatment			−0.01	.968‡		−0.08	.515‡		−0.24	.777‡
EMIC score			0.64	<.001‡		0.36	.003‡		0.53	<.001‡
BUFLS			0.64	<.001‡		0.71	<.001‡		0.67	<.001‡

* Mann Whitney U test, § Kruskal Wallis test, ‡ Spearman Rank correlation with P-scale scores, ** median values not provided as only 1 participant fell into this group.

### Predictors of participation restrictions

Factors significantly contributing to the regression equation were sex, functional limitations (BUFLS), perceived stigma (EMIC), age and lesion size (Table 3). The explained variance of the model was 52%. In the prediction model, females had on average higher (8.1) P-scale scores than men. As functional limitation increases by 1 point, (scale range 0–71), P-sale score increases on average with 0.6 units. As perceived stigma increases by 1 point, (scale range 0–90), P-scale score increases on average with 0.2 units. Having a category II lesion (cross-sectional diameter of 5–15 cm) increases the P-scale score on average with 7.6 points.

**Table 3:** Results of linear regression analyses to statistically predict participation restrictions (n = 132).

Predictors	B	SE	*P*-value	95% CI Lower Bound	95% CI Upper Bound
Constant (reference group is lesion Category I)	−2.1	2.65	.427	−7.36	3.13
Category II	7.6	3.17	.019	1.29	13.82
Category III	5.7	3.72	.126	−1.63	13.10
Sex (female)	8.1	2.43	.001	3.26	12.88
BUFLS	.6	.11	<.001	.35	.78
EMIC	.2	.06	.001	.09	.33
Age*	.2	.09	.073	−.01	.33

Lesion category I: lesions with cross-sectional diameter of less than 5 cm, lesion category II: lesions with 5–15 cm cross-sectional diameter, lesion category III: lesions with >15 cm cross-sectional diameter, on important sites or multiple lesions, BUFLS: functional limitations, EMIC: perceived stigma, * age was centered – 15 years of age.

Post hoc analysis was performed to determine predictive value of lesion size as dichotomized variable (category I vs category II and III) on participation restrictions. Factors significantly contributing to the regression equation were similar as shown in Table 3 as well as the explained variance of the model. Having a category II or III lesion increases the P-scale score by 6.8 points (*P* = .010).

## Discussion

We showed persisting participation restrictions in almost half of the former patients with BU in Ghana and Benin. The percentage of former patients with BU with participation restrictions is less compared to previous studies among former leprosy patients positively screened for difficulties in functioning in Indonesia (about 60%) [18], but is higher compared to former leprosy patients in Bangladesh (34%) [17] and Brazil (35%) [16]. Most commonly reported problems as indicated by former BU patients related to employment. This is in line with a previous study on participation restrictions using the P-scale among a total of 20 leprosy affected persons in Nigeria [14] reporting problems in areas related to work, domestic life and interpersonal relations. And a study among recently diagnosed leprosy patients in India showed that many respondents experience restrictions in areas related to work [31]. Finally the results of our study confirm qualitative findings reporting that BU may cause problems with employment [20]. The other areas in which former BU patients experienced restrictions differed between Ghana and Benin. Predictors of participation restrictions were sex, perceived stigma, functional limitations and the size of the lesion. Women were more at risk for participation restrictions, which may be explained by sociocultural perception differences on participation restrictions between men and women. Further the difference can be explained by a different experience of the negative attitudes of community members as indicated in a previous study [21]. Furthermore it is plausible that women have more tasks and relationships as compared to men, however in Indonesia no difference in participation restrictions between men and women was found [18]. Patients affected by larger lesions may lose more muscle or joint function and as a result are more restricted in participation. In addition functional limitations may also affect people's mobility to participate in their community. Finally feeling stigmatized as a result of being a former BU patient may prevent people to interact with others in and outside the community or participate in relationships.

To our knowledge, this study was the first to use a prediction model for participation restrictions among former patients with BU as measured with the P-scale. Surprisingly duration between end of treatment and time of interview did not influence participation restrictions. In addition participation restrictions were not significantly different for category III lesions compared to category I lesions. It is plausible that the small sample size of former patients with BU with category III lesions resulted in this outcome. Therefore we performed a post hoc analysis dichotomizing small lesions (category I) and large lesions (category II and III). The results of this analysis showed that having a category II or III lesion increases the P-scale score by 6.8 points.

To establish cut-off scores the 95^th^ percentile of the community scores was calculated. Two healthy community controls from Ghana were removed for this analysis because they presented extreme outliers, affecting cut-off tremendously (29 *versus* 14). The cut-offs varied slightly for Ghana and Benin (18 *versus* 14) indicating heterogeneity across countries. Though, we decided to calculate 1 cut-off as preferred for future use in the field.

Several study limitations should be mentioned. The groups of BU patients in Ghana and Benin were heterogeneous as many factors such as case finding activities and exclusion of potential participants due to participation in another study were beyond our control. As a result, former patients in Ghana were treated much more recently and mainly had category I lesion. Furthermore differences regarding employment related problems were found. However background information regarding these differences is not available as it was not the focus of our study, also because we did not expect these differences. In Benin, we aimed for random sampling of the potential participants, however, logistical reasons led to the decision for a convenience sample in certain villages. It is conceivable that selection bias may have occurred. Furthermore, the cross-sectional design of the study prohibits drawing causal relationships. As such, some of the statistical predictors (perceived stigma and functional limitations) of participation restrictions may also be a result of participation restrictions. This is in line with the ICF model encompassing all the dimensions of disability, showing solely bidirectional associations. Finally, due to unknown reasons visible deformity was filled out less frequently leading to missing data and its influence on the P-scale could therefore not be analyzed in the linear regression.

To conclude, we have shown persisting participation restrictions among former patients with BU, even long after treatment had finished and wounds had healed. Unfortunately the introduction of the antibiotic treatment in 2005 has not been able to prevent long-term consequences on the capability to participate in the community. The results indicate active case finding is required, as former patients with BU that presented with small lesions experienced less participation restrictions. POD programs, including stigma reduction strategies and physical and social rehabilitation are needed even after ‘successful’ completion of medical treatment. Such programs should pay extra attention to work integration. Before the development of these POD programs mixed methods studies should be performed to study local meanings of participation restrictions.

## Supporting Information

Checklist S1 STROBE checklist. ** √  =  what is described in the manuscript, NA =  Not applicable.
(DOC)
