# Correction: Persisting Social Participation Restrictions among Former Buruli Ulcer Patients in Ghana and Benin

**DOI:** 10.1371/journal.pntd.0003418

**Published:** 2014-12-01

**Authors:** The PLOS Neglected Tropical Diseases Staff

The thirteenth author’s name is spelled incorrectly. The correct name is: Ymkje Stienstra.
